# Prognosis from Pixels: A Vendor-Protocol-Specific CT-Radiomics Model for Predicting Recurrence in Resected Lung Adenocarcinoma

**DOI:** 10.3390/cancers18020200

**Published:** 2026-01-08

**Authors:** Abdalla Ibrahim, Eduardo J. Ortiz, Stella T. Tsui, Cameron N. Fick, Kay See Tan, Binsheng Zhao, Michelle Ginsberg, Lawrence H. Schwartz, David R. Jones

**Affiliations:** 1 Department of Radiology, Memorial Sloan Kettering Cancer Center, New York, NY 10065, USAzhaob1@mskcc.org (B.Z.);; 2 Department of Surgery, Memorial Sloan Kettering Cancer Center, New York, NY 10065, USA; 3 Department of Epidemiology and Biostatistics, Memorial Sloan Kettering Cancer Center, New York, NY 10065, USA

**Keywords:** lung cancer, radiomics, harmonization

## Abstract

Background: Radiomics can provide quantitative descriptors of tumor phenotype, but translation is often limited by feature instability across scanners and protocols. We aimed to develop and internally validate a protocol-specific CT-radiomics model using preoperative imaging to predict 5-year recurrence in patients with stage I lung adenocarcinoma after complete surgical resection. Methods: The retrospective study included 270 patients with completely resected stage I lung adenocarcinoma from January 2010–December 2021, among whom 23 (8.5%) experienced recurrence within five years. Radiomic features were extracted from routine preoperative CT scans. After preprocessing to remove highly constant and highly correlated features, the Synthetic Minority Over-sampling Technique addressed class imbalance in the training set. Recursive Feature Elimination identified the most predictive radiomic features. An XGBoost classifier was trained using optimized hyperparameters identified through RandomizedSearchCV with cross-validation. Model performance was evaluated using the ROC curve and predictive metrics. Results: Five radiomic features differed significantly between recurrence groups (*p* = 0.007 to <0.001): Shape Sphericity, first-order 90Percentile, GLCM Autocorrelation, GLCM Cluster Shade, and GLDM Large Dependence Low Gray Level Emphasis. The radiomics model showed excellent discriminatory ability with AUC values of 0.99 (95% CI: 0.98–1.00), 0.97 (95% CI: 0.91–1.00), and 0.96 (95% CI: 0.85–1.00) on the training, validation, and test sets, respectively. On the test set, the model achieved sensitivity of 100% (95% CI: 51–100%), specificity of 94% (95% CI: 81–98%), PPV of 67% (95% CI: 30–90%), NPV of 100% (95% CI: 90–100%), and overall accuracy of 95% (95% CI: 83–99%). Conclusions: Under protocol-homogeneous imaging conditions, CT radiomics accurately predicted recurrence in patients with completely resected stage I lung adenocarcinoma. External multi-vendor validation is needed before broader deployment.

## Simple Summary

Radiomics is often limited by poor reproducibility across scanners and acquisition settings, which can reduce predictive performance. In this study, we investigated whether a CT-radiomics signature derived from a standardized CT image acquisition during routine preoperative evaluation can predict recurrence after complete resection of stage I lung adenocarcinoma while controlling for imaging protocol. Our goal is twofold: (1) to provide a practical framework for developing reproducible radiomic signatures Within a uniform image acquisition protocol, and (2) to demonstrate a protocol-specific model that could help identify patients who may benefit from closer surveillance or consideration of additional therapy.

## 1. Introduction

Lung cancer remains a major global health burden and the leading cause of cancer-related deaths worldwide. Non-small-cell lung cancer (NSCLC) accounts for approximately 85% of lung cancers, with lung adenocarcinoma (LUAD) as the predominant histological subtype, comprising nearly half of NSCLC diagnoses [1]. Due to the advancement in screening programs, diagnoses of stage I NSCLC have increased from 23.5% in 2010 to 29.1% in 2017 [2]. Complete surgical resection remains the standard of care for patients with stage I NSCLC. However, 10–30% of patients with completely resected stage I NSCLC develop recurrence and experience reduced overall survival [3]. Because scanner and protocol variability significantly affects the reproducibility of quantitative features, this study deliberately examines a single-vendor (GE), fixed-protocol preoperative CT cohort; we acknowledge up front that this design prioritizes internal validity over broad generalizability, and we justify its clinical relevance for centers with standardized imaging pathways. It also serves as a framework for developing new robust tools.

Using TNM clinical staging systems alone to guide treatment poses inherent limitations and inconsistencies in reliably predicting recurrence risk at an individual patient level [4]. Emerging evidence highlights substantial intra-stage heterogeneity, with patients sharing identical clinical stages demonstrating markedly different recurrence outcomes [5]. Tumor radiographic (e.g., higher SUVmax), histologic (e.g., lymphovascular invasion [6], genomic features (e.g., alterations in SMARCA4, TP53) [7], and molecular markers (e.g., presence of circulating tumor DNA after resection) [8] showed promise in identifying patients with resected stage I NSCLC patients at high risk of recurrence. These data underscore the need for precise, individualized tools to stratify recurrence risk and optimize postoperative management.

Conventional imaging interpretation relies heavily on qualitative visual assessment, which can be subjective and insufficient for capturing subtle yet clinically relevant tumor characteristics predictive of recurrence [9,10]. The field of radiomics uses high-throughput computational algorithms to extract and analyze quantitative features from medical images, transforming routine clinical images into mineable data representing tumor biology and heterogeneity [11]. Radiomic features describe morphological, textural, and intensity-based characteristics that are imperceptible to the naked eye, reflecting underlying tumor phenotypic traits such as tumor heterogeneity, growth patterns, and microenvironmental alterations [10,12].

Numerous studies have demonstrated that radiomics can enhance predictive accuracy for various clinical outcomes in oncology, including survival, therapeutic response, and disease recurrence [12,13]. Specifically in lung cancer, radiomics has been evaluated extensively for predicting prognosis, pathological characteristics, genetic mutations, and treatment responses, showing promising utility as a non-invasive imaging biomarker complementing clinical and pathological information [14,15]. Nevertheless, identifying recurrence-associated radiomic signatures could substantially improve individualized care by flagging high-risk patients for intensified adjuvant therapy and closer surveillance. At the same time, translation is limited by feature instability across scanners and protocols, non-standardized preprocessing/segmentation, contrast enhancement, and heterogeneous modeling and validation [16,17,18,19,20,21]. Because there is no widely validated radiomics-specific harmonization method for cross-vendor deployment, controlling acquisition remains a pragmatic lever to improve feature comparability [22,23,24,25,26,27]. Previous studies indicated that radiomic feature values vary significantly for the same lesion across different scans, depending on the degree of variation in the acquisition and reconstruction parameters. With no currently validated radiomics-specific harmonization methods, attention must be paid to the inclusion criteria of patient scans.

Radiomics-based models are particularly attractive in early-stage lung cancer because routine preoperative CT imaging is obtained in nearly all patients prior to surgery, whereas the reporting of high-risk pathologic features at different hospitals can be more variable, especially for newer entities such as spread through air spaces. Because radiomics leverages preoperative data that are almost universally available, it has the potential to become a powerful, scalable risk-stratification tool for the broader lung cancer population. In this context, our study serves as a pilot effort to test the feasibility of a CT-based radiomics model for predicting recurrence. Consequently, the aim of this study is to investigate the potential of radiomics derived from routine preoperative CT imaging in predicting recurrence after complete surgical resection in patients with stage I LUAD, using a comparability-oriented methodology. This work advances prior studies by (i) focusing on a clinically homogeneous population (pathologic stage I LUAD following curative surgery), (ii) minimizing acquisition-induced variance through a single-vendor (GE), fixed-protocol dataset, and (iii) implementing a design aimed at reducing feature variability. Ultimately, developing and validating a reliable radiomics-driven predictive tool could inform tailored postoperative surveillance and therapeutic strategies, potentially enabling earlier interventions and improving patient outcomes. We recognize that our protocol-constrained design limits immediate cross-vendor generalizability; however, it addresses a realistic first use case—local deployment within standardized imaging environments—and establishes a reproducible baseline for subsequent external validation and harmonization. The signature was developed under tightly controlled conditions, ensuring that any observed limitations are less likely to be attributable to image acquisition variability or vendor-related effects.

## 2. Materials and Methods

### 2.1. Patient Data

This retrospective, single-institution study was approved by the institutional review board and was conducted in compliance with HIPAA. A waiver of written informed consent was provided by the institutional review board.

**Figure 1:**
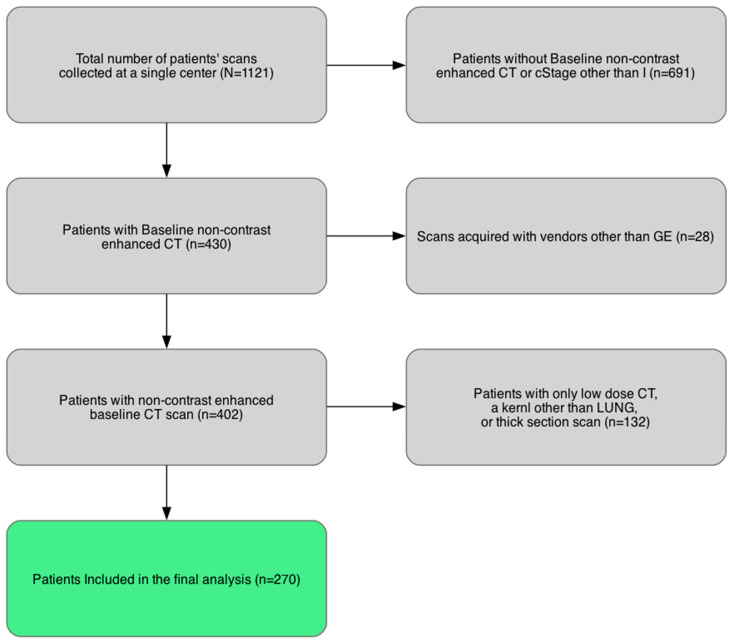
Inclusion and exclusion criteria for the study cohort. Abbreviations: cStage = clinical stage.

A retrospective review of a prospectively maintained thoracic surgery database at a tertiary cancer center was performed. Consecutive patients with clinical stage I histologically proven LUAD who underwent complete surgical resection from January 2010 to December 2021 were identified. A total of 1121 patients were retrieved. The inclusion criteria for the study were as follows: (i) surgical pathology confirmed microscopically margin-negative (R0) resection; and (ii) availability of presurgical non-contrast scans. To address potential limitations in radiomics reproducibility, we restricted the number of patients included in the analysis. Specifically, we selected cases acquired on GE scanners (GE Healthcare, Chicago, IL, USA) —the most common vendor in our dataset—using a LUNG reconstruction kernel and a 1.25 mm slice thickness. Accordingly, a total of 270 patients were included in the final analysis (Figure 1). The acquisition and reconstruction parameters of the included patients are listed in Table 1.

### 2.2. Lesion Segmentation and Radiomics Extraction

**Figure 2:**
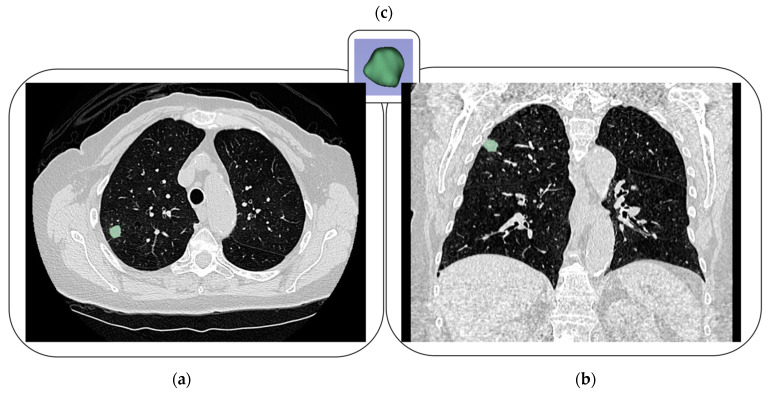
Example tumor segmentation (green) shown on (**a**) axial and (**b**) coronal CT views, with (**c**) a 3D volume rendering.

The lesions were segmented on the CT scans by a research radiologist with 15 years of experience in image segmentation. All segmentations underwent visual quality control for accuracy and consistency (and were corrected when needed) prior to feature extraction. Figure 2 shows an example of lesion segmentations. The CT scans and corresponding 3D lesion segmentations were used to extract radiomic features with the PyRadiomics toolbox (v3.0.1) [22]. All scans were resampled in the axial plane to 1 mm × 1 mm using Cosine Windowed Sinc interpolation, which showed favorable feature stability in prior reproducibility experiments [18]. A fixed bin width of 25 Hounsfield units was used for gray-level discretization, a commonly used setting for CT radiomics and consistent with recommendations to use fixed bin widths for calibrated HU images [21]. No other preprocessing techniques were applied. A total of 105 radiomic features (original features) were extracted from each lesion.

### 2.3. Statistical Analysis

Statistical analysis was conducted using Python (version 3.8). The dataset was split into training (70%, *n* = 189), validation (15%, *n* = 40), and testing (15%, *n* = 41) subsets, with stratification to maintain class distribution using train_test_split from scikit-learn (v1.3.0) [23]. Feature preprocessing included removing highly constant features (identical values in >95% of samples) and removing highly correlated features (Pearson’s r > 0.85) to reduce redundancy.

To address class imbalance in the training set, the Synthetic Minority Over-sampling Technique (SMOTE) was employed with k_neighbors = 5 (default) and applied to the training data only, leaving validation and test sets untouched [24]. Subsequently, feature selection was performed using Recursive Feature Elimination (RFE) with a Random Forest Classifier as the underlying estimator to select the top predictive features [25,26].

An XGBoost classifier was utilized for predictive modeling [16]. Hyperparameter optimization was conducted using RandomizedSearchCV with 1000 iterations and 5-fold stratified cross-validation within the training set, maximizing AUC. The search space included: n_estimators (50–800), max_depth (2–8), learning_rate (0.01–0.3), subsample (0.5–1.0), colsample_bytree (0.5–1.0), min_child_weight (1–10), gamma (0–5), reg_alpha (0–1), and reg_lambda (0.5–5). Model performance was evaluated using AUC and classification metrics (sensitivity, specificity, PPV, NPV, accuracy) computed from the confusion matrix. Confidence intervals (95%) for AUC values were determined via bootstrap resampling with 1000 iterations. The model was tuned using the validation set, and the best-performing model was assessed on the held-out test set.

Additionally, the Wilcoxon rank-sum test was applied to compare differences in selected radiomic features between patients with and without recurrence, facilitating interpretation of feature relevance. All tests were two-tailed, with statistical significance set at *p* < 0.05. Data analysis scripts were implemented using open-source libraries, including scikit-learn (1.3.0), XGBoost (3.0.0), pandas (2.0.3), numpy (1.23.5), matplotlib (3.7.2), and scipy (1.11.1).

## 3. Results

### 3.1. Patient Characteristics and Recurrence Rates

A total of 270 patients (mean age, 69.4 ± 8.5; 93 males and 177 females) were included in the analysis. Of these patients, 23 (8.5%) experienced recurrence within 5 years. Baseline demographic and clinical characteristics of the overall cohort are summarized in Table 2; with the different groups’ characteristics reported separately.

### 3.2. Feature Selection and Model Development

After feature preprocessing and selection, five radiomic features were included in the final predictive model. The features selected by RFE included: shape Sphericity, first-order 90Percentile, gray-level cooccurrence matrix (GLCM) Autocorrelation, GLCM Cluster Shade, gray-level dependence matrix (GLDM) Large Dependence Low Gray Level Emphasis. These features showed significant differences between the recurrence groups, with Wilcoxon rank-sum test *p* values ranging from 0.007 to < 0.001.

### 3.3. Model Performance

**Figure 3:**
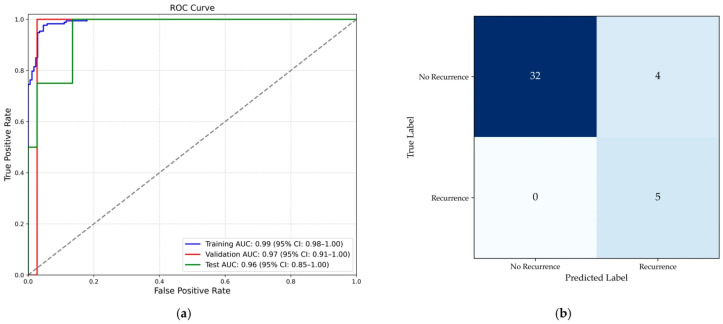
(**a**) ROC curves showing model performance on the training, validation, and test sets; (**b**) confusion matrix on the test set. Abbreviations: AUC, area under the receiver operating characteristic curve.

The radiomics-based model demonstrated robust performance in predicting recurrence. The model achieved an AUC of 0.99 (95% CI: 0.98–1.00) on the training set, 0.97 (95% CI: 0.91–1.00) on the validation set, and 0.96 (95% CI: 0.85–1.00) on the testing set (Figure 3a). At the optimal thresholds, the model’s predictive performance metrics on the testing set were as follows: sensitivity of 100% (95% CI: 51–100%), specificity of 94% (95% CI: 81–98%), PPV of 67% (95% CI: 30–90%), NPV of 100% (95% CI: 90–100%), and overall accuracy of 95% (95% CI: 83–99%) (Figure 3b).

## 4. Discussion

Patients with early stage LUAD have variable rates of recurrence which cannot be predicted accurately based upon the traditional tumor-node-metastasis staging criteria. This prompted us to test whether standardized preoperative CT radiomics could improve individual risk prediction after curative surgery. In this retrospective, single-center analysis of 270 stage I patients, we intentionally restricted imaging heterogeneity to a single vendor and protocol (GE, non-contrast, LUNG kernel, 1.25 mm) to prioritize feature reproducibility given known protocol sensitivity [23,24,28,29,30]. Twenty-three of 270 (8.5%) had recurrence within five years. A five feature radiomics model—Shape Sphericity, first order 90th Percentile, GLCM Autocorrelation, GLCM Cluster Shade, GLDM Large Dependence Low Gray Level Emphasis—predicted recurrence with test set AUC 0.96, sensitivity 100%, specificity 94%, and accuracy 95% (versus chance AUC 0.50; *p* < 0.001).

The significant differences in the selected features between recurrent and non-recurrent groups underscore their potential to quantitatively characterize tumor aggressiveness and heterogeneity. Shape Sphericity, for instance, provides critical insights into tumor morphology, and has been previously associated with invasive behavior and poor prognosis in lung cancer [31,32]. Texture features derived from the GLCM, such as Autocorrelation and Cluster Shade, quantify subtle textural differences reflecting tumor microenvironment complexity, vascularity, and hypoxia. Indeed, previous studies highlighted GLCM features as promising quantitative imaging biomarkers associated with aggressiveness and recurrence in various malignancies, including lung cancer [12,33]. Similarly, GLDM Large Dependence Low Gray Level Emphasis captures specific low-intensity regions within the tumor, potentially reflecting necrotic or hypoxic areas, which are known to predict poor outcomes and recurrence [34,35]. First-order features describe the distribution of Hounsfield unit values within the ROI. First-order 90Percentile is the 90th percentile Hounsfield unit value within the ROI. Higher values correspond to higher solidity or density of the tumor.

The decision to analyze a vendor/protocol–specific cohort is grounded in quantitative methodology work showing that many handcrafted radiomic features (HRFs) vary substantially with image acquisition and reconstruction. In phantom analyses where only in-plane resolution differed, pairwise concordance (CCC > 0.90) for the same object ranged from ~43% to ~95%; windowed-sinc resampling increased, but did not guarantee, agreement, and adding ComBat provided limited additional benefit [28]. Complementarily, across 31,375 CT pairs, the convolution kernel was the dominant driver of reproducibility (48% importance), followed by slice thickness (33%) and pixel spacing (19%). When those three resolution parameters matched (MaasPenn > 0.98), the probability that ≥90% of HRFs were reproducible was 0.97 (false alarm 3%); when mismatched (score < 0.75), ≤10% were reproducible with probability 0.74 (false alarm 19%) [23]. Because no radiomics-specific harmonization tool has yet been validated for routine CT use, limiting protocol heterogeneity at the source is a defensible and novel design choice that maximizes the pool of reproducible features and minimizes protocol-driven confounding [23,28]. In line with prior evidence favoring windowed-sinc interpolation for reproducibility, we also standardized in-plane sampling to 1 mm × 1 mm using Cosine Windowed Sinc (CWS) [28]. To our knowledge, this is the largest protocol-homogeneous CT radiomics dataset analyzed for postoperative LUAD recurrence to date, underscoring the potential of protocol-specific signatures.

Notably, the final radiomic features identified through RFE demonstrated robust significance, with Wilcoxon rank-sum test *p* values < 0.01. This rigorous statistical approach, combined with subsequent machine-learning optimization, substantially enhanced the robustness and performance of the model. The discriminative ability of the radiomics-based model was exceptionally high, even in the independent testing cohort. These results indicate strong reproducibility and predictive accuracy, reinforcing the clinical potential of radiomics as a complementary prognostic tool. The performance metrics further underscore the clinical utility of the model. Particularly, the high sensitivity and NPV offer substantial clinical benefit in reliably identifying patients at low risk for recurrence. This high NPV may help clinicians confidently exclude low-risk patients from unnecessary aggressive surveillance and consideration of adjuvant systemic treatment, thus reducing healthcare resource burdens and patient anxiety. Nevertheless, it should be interpreted with caution due to the relatively small sample size. Importantly, maintaining protocol uniformity during model development likely helped preserve true biological signal in texture features that are otherwise vulnerable to kernel/thickness/spacing effects.

In a postoperative stage I population with a relatively low recurrence prevalence, even a highly specific model will generate some false-positive (FP) predictions, which can lower positive predictive value (PPV). At our chosen operating point, the model was tuned to prioritize sensitivity, because false-negative (FN) predictions (i.e., incorrectly labeling a patient as low risk) could delay recurrence detection and reduce opportunities for earlier intervention or trial enrollment. In contrast, FP predictions primarily risk increased imaging intensity (e.g., shorter-interval chest CT, earlier PET/CT, or closer clinical follow-up), with potential downsides including additional cost, radiation exposure, and patient anxiety. These potential harms are best mitigated by using the radiomics score as an adjunct to established clinicopathologic risk factors (e.g., lymphovascular invasion, visceral pleural invasion, STAS) rather than as a stand-alone trigger for adjuvant therapy.

Compared to conventional staging systems and clinical prognostic indicators, radiomics provides an objective and quantitative approach to assess tumor biology, offering additional prognostic insights beyond traditional clinical and pathological assessments. The current TNM staging system relies heavily on anatomical and pathological criteria, which, despite significant utility, cannot fully capture intra-stage heterogeneity and subtle biologic differences underlying patient outcomes [4,36]. Our findings strongly suggest that radiomics-derived quantitative imaging biomarkers could address these gaps by providing personalized, tumor-specific biological information that is otherwise inaccessible through standard imaging interpretation or clinical evaluations alone.

In terms of methodology, our rigorous analytical pipeline included careful data preprocessing, feature normalization, robust feature selection through RFE, and class imbalance correction via SMOTE, enhancing the reliability and validity of the findings. Furthermore, our approach incorporated extensive hyperparameter optimization using randomized search cross-validation for XGBoost classifier, effectively reducing potential overfitting and enhancing model generalizability [25]. Methodologically, aligning acquisition/reconstruction and then using CWS resampling is consistent with best-available evidence for maximizing feature agreement when pixel spacing differs [28], and our emphasis on protocol-specific training provides a practical path toward clinical robustness while radiomics-specific harmonization tools mature [23]. It also serves as novel framework for developing robust radiomic signatures.

Nevertheless, our study has limitations. First, the duration of follow-up duration varied significantly across patients, and therefore, additional patients may experience recurrence with further follow-up. Second, although we report internal validation performance metrics, external validation in multi-institutional diverse patient cohorts remain essential to confirm generalizability of the model. Third, the relatively small number of recurrence events (8.5%) in our cohort, though consistent with literature rates, could affect the statistical precision of some performance metrics, particularly PPV. Larger cohorts with higher event frequencies could further refine estimates and strengthen confidence in predictions. Finally, by prioritizing internal validity through vendor/protocol restriction, we reduced the sample size and limited immediate generalizability of the model. Nevertheless, protocol-specific signatures might counteract the lack of proper imaging harmonization methods. Furthermore, this methodology provides a rapidly translatable framework, allowing other institutions to easily develop institution-specific, protocol-standardized radiomics signatures.

## 5. Conclusions

This study demonstrates the potential of protocol-homogeneous CT radiomics to predict recurrence following complete surgical resection in patients with stage I lung adenocarcinoma. We present a vendor- and protocol-specific radiomic signature that accurately predicts 5-year recurrence under controlled imaging conditions. Future work should include external, multi-institution validation and assessment of harmonization strategies before broader clinical deployment.

## Figures and Tables

**Table 1:** Acquisition and reconstruction parameters of included scans.

Vendor	Model	X-Ray Tube Current (mA)	Exposure Time (ms)	kVP	Reconstruction Kernel	Slice Thickness (mm)	Pixel Spacing (mm^2^)
GE Medical Systems	Discovery; LightSpeed; Revolution	118–383	500–700	120, 140	LUNG	1.25	0.55 × 0.55–0.98 × 0.98

**Table 2:** Patient characteristics.

Number of Patients	No Recurrence (*n* = 247)	Recurrence (*n* = 23)
Age (mean, years) ± standard deviation	69.1 ± 8.6	72.7 ± 7.3
Biological sex		
Male	84 (34.0%)	9 (39.1%)
Female	163 (66.0%)	14 (61.9%)
Time to recurrence (Median, interquartile ranges, in days)		762 (331–954)
Smoker		
Current	21 (8.5%)	4 (17.4%)
Former	169 (68.4%)	14 (60.9%)
Never	57 (23.1%)	5 (21.7%)
Visceral pleural invasion		
Yes	15 (6.1%)	5 (21.7%)
No	232 (93.9%)	18 (78.3%)
Lymphovascular Invasion		
Yes	43 (17.4%)	10 (43.5%)
No	204 (82.6%)	13 (56.5%)
Spread through airspaces (STAS)	*n* = 215	*n* = 16
Yes	87 (40.5%)	12 (75.0%)
No	128 (59.5%)	4 (25.0%)
SUV_max_	2.7 ± 3.7	4.0 ± 3.3
Clinical stage		
IA1	25 (10.1%)	2 (8.7%)
IA2	147 (59.5%)	16 (69.6%)
IA3	61 (24.7%)	4 (17.4%)
IB	14 (5.7%)	1 (4.3%)
Pathologic stage		
IA1	79 (32.0%)	3 (13.0%)
IA2	111 (44.9%)	9 (39.2%)
IA3	32 (13.0%)	5 (21.7%)
IB	25 (10.1%)	6 (26.1%)
Surgical procedure		
Lobectomy	127 (51.4%)	8 (34.8%)
Segmentectomy	56 (22.7%)	5 (21.7%)
Wedge	64 (25.9%)	10 (43.5%)

Abbreviations: SUV_max_, maximum standard uptake value.

## Data Availability

Data generated or analyzed during the study are available from Binsheng Zhao (zhaob1@mskcc.org).
